# Risk Factors for Acute Acquired Comitant Esotropia in Children and Young Adults: A Systematic Review

**DOI:** 10.22599/bioj.386

**Published:** 2024-09-03

**Authors:** Manjushri Yuan Rou Lee, Mei Shi Pearl Lee

**Affiliations:** 1 Orthoptics Department, Singapore National Eye Centre, Singapore

**Keywords:** acute acquired comitant esotropia, risk factors, strabismus, children, young adults

## Abstract

**Background:**

Acute acquired comitant esotropia (AACE) is a rare subtype of esotropia that occurs after infancy. The exact pathogenesis of AACE remains unknown with aetiologies ranging from benign conditions to serious underlying neurological diseases being reported. Given the elusive characteristic of AACE, diagnostic and management guidelines remain unclear. This systematic review aims to contribute to this field by summarising the risk factors for AACE reported thus far.

**Methods:**

A systematic review was conducted with papers found in CINAHL, MEDLINE, Cochrane library, PubMed databases and other sources. Eligible studies investigating the risk factors for, and clinical features of, AACE in children and young adults were critically appraised before relevant data were extracted and discussed via a narrative summary.

**Results:**

Twelve studies were included in the final review, of which six and eight papers reported on benign and non-benign risk factors for AACE respectively. Identified benign risk factors varied among studies, while non-benign risk factors were associated with intracranial pathologies, multiple sclerosis and head trauma.

**Conclusion:**

Given the low generalisability of study findings, no definitive conclusions can be drawn on the significance of each risk factor on AACE development. Further prospective research with more objective measurements of ‘near work’, larger sample sizes and control groups is required to better ascertain any cause-effect relationship, refine the diagnostic criteria for each AACE subtype and advise on appropriate management guidelines for AACE.

## Introduction

Acute acquired comitant esotropia (AACE) is a type of esotropia marked by the onset of diplopic comitant esotropia after infancy (Lekskul et al., 2021). Three distinct AACE subtypes, each named after the doctor who first identified them, were originally described by Burian and Miller (1958): Type 1 (Swan) results from binocular disruption secondary to monocular occlusion; Type 2 (Franceschetti) is non-accommodative, often associated with mild hypermetropia and may be affected by physical or mental stress; Type 3 (Bielschowsky) is related to uncorrected myopia and prolonged near work in older children and adults. Additional subclassifications of decompensated, neurologic, cyclic and secondary AACE were recently suggested by Buch and Vinding (2015). However, reports supporting these recommendations remain limited in the current literature (Lekskul et al., 2021).

While AACE was reported to constitute 0.3% of all childhood strabismus, incidence amongst young adults and the general population remains unknown as AACE cases are rare (Lekskul et al., 2021; Mohney, 2007). Despite its low prevalence rate, the disease burden of AACE on individuals and the society is significant (Buffenn, 2021). Patients with binocular inhibition secondary to strabismus were found to have reduced functional vision and quality of life (Tandon et al., 2014), especially younger children who have weaker coping mechanisms to negative peer attitudes (Sim, Yap and Chia, 2014). Without proper treatment, AACE poses increased risks of injury, irreversible vision loss and decreased functional abilities, ultimately reducing one’s socioeconomic contribution significantly (Buffenn, 2021).

Though AACE has been associated with serious underlying neurological diseases such as brain tumours, hydrocephalus and Arnold-Chiari Type 1 malformation (Hoyt and Good, 1995; Liu et al., 1997), most cases stem from benign conditions of binocular dysfunction and refractive errors (Lekskul et al., 2021). Recent studies also hypothesised that prolonged excessive near work, including reading, writing and viewing of electronic devices could be a risk factor for AACE (Meng et al., 2022; Sefi-Yurdakul, 2022). The increased use of digital devices for home-based learning during the COVID-19 outbreak, in particular, was claimed to disrupt the balance of extraocular muscle tones and motor fusion mechanisms, leading to the onset of AACE (Yabanoğlu and Şekeroğlu, 2022). Depending on the aetiology of AACE, treatment could involve glasses, prisms, botulinum injection, strabismus surgery, neurosurgical decompression or a combination of these to resolve troublesome symptoms and restore binocular functions (Gilbert, Koo and Heidary, 2017).

Given the elusive pathophysiology of AACE, thorough evaluation of all patients via detailed history taking and orthoptics examination is required (Hoyt and Good, 1995). The need for neuroradiological investigation, however, is debatable with some studies ordering these tests only when associated neurological signs were present and other studies performing neuroimaging on all patients with AACE to exclude any intracranial pathology (Dotan, Keshet and Friling, 2020; Gilbert, Koo and Heidary, 2017; Kim and Noh, 2021; Turan and Kansu, 2016).

Currently, no systematic review analysing the risk factors for, and clinical characteristics of, AACE has been conducted and management guidelines for AACE remain unclear. This study hence aims to contribute to this field by summarising the various benign and non-benign risk factors of AACE reported thus far while describing the incidence, associated clinical characteristics, proposed treatment options and prognosis for each type of AACE in children and young adults, allowing clinicians to better advise on preventive measures and decide on the most appropriate diagnostic tests and treatment for patients with AACE.

## Methods

### Eligibility criteria

#### Types of participants

Studies involving children (aged 1 to 17) and/or young adults (aged 18 to 35) diagnosed with any type of AACE were included in this review. Participants with a previous history of strabismus and/or incomitant esotropia were excluded.

#### Types of studies

All prospective and retrospective cohort studies, case-control studies, case series and case reports which have documented the clinical histories, relevant measurements of esotropia and refractive errors, treatment and outcomes of patients with AACE were included in this review.

#### Exclusion criteria

- Studies without clear diagnostic criteria for AACE and its subtypes.
- Studies where the comitancy of esotropia was not made explicit.
- Studies where neuroimaging was not performed on all patients to determine if any underlying neurological condition was present.
- Studies proposing excessive near work as a risk factor but without a clear definition or description of ‘near work’.
- Studies lacking follow-up or with a follow-up period of less than a year.

With regards to case series, papers were included when at least half of the reported patients met the above eligibility criteria. Only clinical information from eligible patients were considered in the final analysis.

#### Type of exposure

Risk factors for AACE were categorised into benign and non-benign in this review.

1. Benign includes, and is not limited to, monocular deprivation, uncorrected refractive error, stress and excessive near work in the absence of underlying neurological conditions.
2. Non-benign includes those of neurological origin.

#### Types of outcome measures

The primary outcome measure was the diagnosis of AACE and its type. Secondary outcomes included incidence among the study population, clinical characteristics, management and prognosis of each type of AACE.

### Identification of studies

A comprehensive literature search on CINAHL, MEDLINE, Cochrane library and PubMed databases was performed between September 5th and 30th, 2022. Additional sources of information were screened for in Google Scholar, the Australian Orthoptic Journal and reference lists of included studies. While the date and geographical subset of publication were not restricted to ensure the widest coverage possible, full-text articles had to be available in English.

The search for eligible publications was conducted independently by the authors (PL and ML) to ensure reproducibility and transparency of the results. A summary of the literature search strategy is provided in Table 1.

**Table 1:** Summary of literature search strategy.


1	‘Child*’ OR ‘Paediatric’ OR ‘Teenager’ OR ‘Young child*’ OR ‘Young adult*’ OR ‘Young patient*’ OR ‘Adolescent*’ OR ‘Young person’ OR ‘Young people’

2	‘?etiolog*’ OR ‘Cause*’ OR ‘Risk factor*’ OR ‘Characteristic*’ OR ‘Classification*’ OR ‘Feature*’ OR ‘Finding*’ OR ‘Associat*’ OR ‘Risk*’ OR ‘Neuro*’ OR ‘Tumo?r’ OR ‘Malformation’ OR ‘Benign’ OR ‘Excessive’ OR ‘Accommodati*’ OR ‘Myopi*’ OR ‘Non?Benign’

3	‘Acute acquired comitant esotropia’ OR ‘AACE’ OR ‘Acquired comitant esotropia’ OR ‘Acute comitant esotropia’ OR ‘Acute acquired concomitant esotropia’ OR ‘Acquired concomitant esotropia’ OR ‘Acute concomitant esotropia’ OR ‘Acute onset esotropia’ OR ‘Comitant esotropia’ OR ‘Concomitant esotropia’ OR ‘Acquired esotropia’

4	#1 AND #2 AND #3

5	#1 AND #3

6	#2 AND #3

7	#4 OR #5 OR #6

Filter: Human, English Language	


Legend: * = To include variant endings of a root term, ? = To substitute one or more letters of a word.

### Study selection

All search results were exported to citation management software EndNote X9. Duplicate studies were first removed before the titles and abstracts of all collated studies were assessed for relevance. Full-text articles of studies that appeared eligible were then obtained through La Trobe University Library and the Interlibrary Loan and Document Delivery Service for further evaluation based on the selection criteria listed above. The entire selection process was conducted independently by both authors with discrepancies resolved via discussion. Studies whereby consensus was not reached were excluded in this review.

### Critical appraisal

The quality of included studies was assessed using the appropriate Joanna Briggs Institute’s (JBI) checklist for cohort studies, case-control studies, case series or case reports (Porritt, Gomersall and Lockwood, 2014). The presence of bias for each criterion was reviewed independently by both authors and disagreements were resolved via discussion. An overall evaluation for each study was then made and only studies with a low risk of bias were included in the final review.

### Data extraction

One author, PL, collected and collated the following information from each included study using a standardised Microsoft Excel spreadsheet:

- Study identification: Authors, date of publication
- Study design: Type of study, clinical setting, follow-up duration
- Participant characteristics: Sample size, age, gender, refractive error (if any), type of AACE diagnosed with
- Identified risk factor(s) for AACE
- Incidence, clinical characteristics (including any relevant measurements), treatment and prognosis for each type of AACE

### Data synthesis

The study selection process was presented using a Preferred Reporting Items for Systematic Reviews and Meta-Analysis (PRISMA) flow diagram (Page et al., 2021). The overall quality and study characteristics of each paper were summarised in different tables.

Additionally, two separate tables for benign and non-benign risk factors were used to compile data on the primary and secondary outcomes extracted from each study. Findings of this review were then discussed and contextualised via a narrative summary.

## Results

### Search results

**Figure 1:**
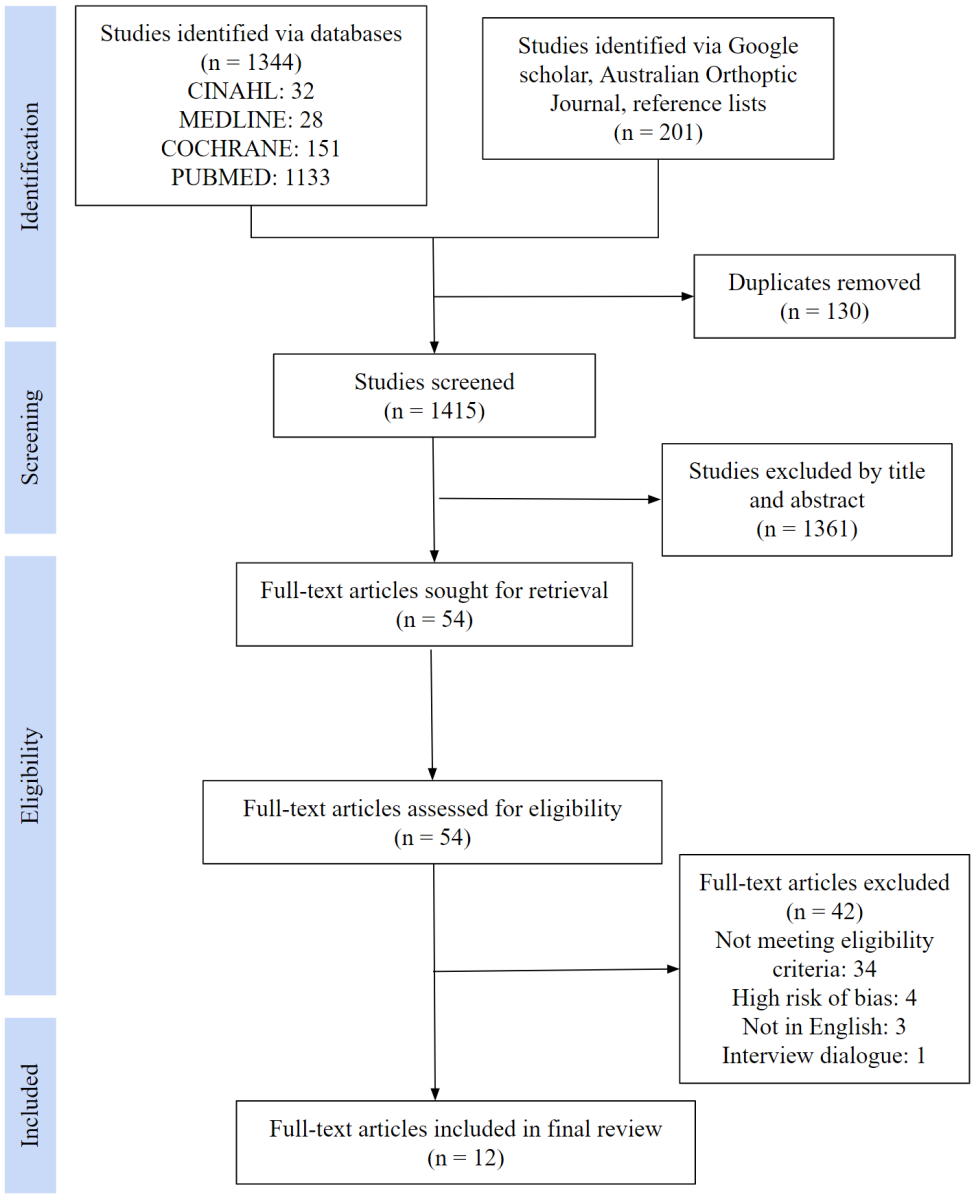
PRISMA study selection flow diagram.

One thousand, three hundred and forty-four articles were identified via the four databases and 201 articles were obtained from other sources. After removing the 130 duplicates, 1415 abstracts and titles were screened for relevance by both authors. Of these, 54 full-text articles were retrieved and assessed for eligibility. 42 articles were excluded as 34 did not meet the inclusion criteria, four had high risks of bias, three were not available in English and one was a vague interview transcript. With consensus reached between the authors, 12 full-text articles with a total of 39 study participants were included in the final review. The study selection process is illustrated in Figure 1.

### Study quality

The appropriate JBI checklists for case series (Table 2) and reports (Table 3) were used to assess the quality of the 16 eligible papers (Porritt et al., 2014). Despite being of relatively high quality, Rajavi, Sabbaghi and Abdi (2016) was excluded due to inconsistent reporting of clinical information. Only 12 studies that fulfilled most of the checklist criteria were deemed as having a low risk of bias and were included in the final review, of which four were case series and eight were case reports.

**Table 2:** Summary of JBI’s checklist findings for case series.


AUTHOR(S)	CLEAR INCLUSION CRITERIA	CONDITION MEASURED IN A STANDARD AND RELIABLE WAY	VALID METHODS USED FOR IDENTIFICATION OF CONDITION	CONSECUTIVE INCLUSION OF PARTICIPANTS	COMPLETE INCLUSION OF PARTICIPANTS	CLEAR REPORTING OF DEMOGRAPHICS OF PARTICIPANTS	CLEAR REPORTING OF CLINICAL INFORMATION	CLEAR REPORTING OF OUTCOMES/FOLLOW-UP RESULTS	CLEAR REPORTING OF DEMOGRAPHIC INFORMATION OF CLINIC(S)	APPROPRIATE STATISTICAL ANALYSIS	OVERALL APPRAISAL

Lyons, C.J., Tiffin, P.A.C. & Oystreck, D. (1999)	✓	✓	✓	✓	✓	✓	✓	✓	✓	N/A	Include

Sefi-Yurdakul, N. (2022)	✓	✓	✓	✓	✓	✓	✓	✓	**×**	N/A	Include

Simon, A L. & Borchert, M. (1997)	✓	✓	✓	✓	✓	✓	✓	✓	✓	N/A	Include

Turan, E.K. & Kansu, T. (2016)	✓	✓	✓	✓	✓	✓	✓	✓	**×**	N/A	Include

Yilmaz, T.P., Fatihoglu, U. & Sener, E.C. (2020)	✓	Unsure	Unsure	✓	✓	**×**	**×**	**×**	**×**	N/A	Exclude

Zakher, M., Simon, J.W. & Zobal-Ratner, J. (2019)	✓	Unsure	✓	✓	✓	**×**	**×**	**×**	**×**	N/A	Exclude


**Table 3:** Summary of JBI’s checklist findings for case reports.


AUTHOR(S)	CLEAR DEMOGRAPHIC CHARACTERISTICS	CLEAR DESCRIPTION AND PRESENTATION OF PATIENT’S HISTORY AS A TIMELINE	CLEAR DESCRIPTION OF CURRENT CLINICAL CONDITION OF PATIENT ON PRESENTATION	CLEAR DESCRIPTION OF DIAGNOSTIC TESTS AND RESULTS	CLEAR DESCRIPTION OF TREATMENT	CLEAR DESCRIPTION OF POST-INTERVENTION CLINICAL CONDITION	IDENTIFICATION AND DESCRIPTION OF ADVERSE/UNANTICIPATED EVENTS	TAKEAWAY LESSON(S)	OVERALL APPRAISAL

Armenti, S.T., Miller, J M.L., Gomez-Hassan, D., Gappy, C. & Cornblath, W.T. (2021)	✓	✓	✓	✓	✓	✓	N/A	✓	Include

Astle, W. & Miller, S.J. (1994)	✓	✓	✓	✓	✓	✓	N/A	✓	Include

Biousse, V., Newman, N.J., Petermann, S.H. & Lambert, S.R. (2000)	✓	✓	✓	✓	✓	✓	N/A	✓	Include

Defoort-Dhellemmes, S., Denion, E., Arndt, C.F., Bouvet-Drumare, I., Hache, J. & Dhellemmes, P. (2002)	✓	✓	✓	✓	✓	✓	N/A	✓	Include

Fukuo, Y., Abe, T. & Hayasaka, S. (1998)	✓	✓	✓	✓	✓	✓	✓	✓	Include

Hussaindeen, J.R., Mani, R., Agarkar, S., Ramani, K.K. & Surendran, T.S. (2014)	✓	✓	✓	✓	✓	✓	✓	✓	Include

Kang, K.D., Kang, S.M. & Yim, H.B. (2006)	✓	✓	✓	✓	✓	✓	✓	✓	Include

Lee, J.M., Kim, S.H., Lee, J.I., Ryou, J.Y. & Kim, S.Y. (2009)	✓	✓	✓	✓	✓	✓	✓	✓	Include

Lewis, A.R., Kline, L.B. & Sharpe, J.A. (1996)	**×**	**×**	✓	×	**×**	✓	N/A	✓	Exclude

Rajavi, Z., Sabbaghi, H. & Abdi, S. (2016)	✓	✓	**×** Comitancy of strabismus mentioned in abstract but not demonstrated within full-text article.	✓	✓	✓	N/A	✓	Exclude


### Study characteristics

With reference to Table 4, the publication years of all included studies ranged from 1994 to 2022. While three case series were retrospective and one was prospective, all case reports were retrospective in nature. Only five articles mentioned a study location, which included the United States, Korea, Denmark and Turkey. Follow-up duration across all papers ranged between 12 months to nine years.

**Table 4:** Summary of study characteristics.


AUTHOR(S)	YEAR OF PUBLICATION	STUDY TYPE	STUDY LOCATION	FOLLOW-UP DURATION

Sefi-Yurdakul, N.	2022	Retrospective case series	Turkey	12 to 64 months
	
Simon, A.L. & Borchert, M.	1997		United States	12 to 24 months
	
Turan, E.K. & Kansu, T.	2016		–	1 to 9 years

Lyons, C.J., Tiffin, P.A.C. & Oystreck, D.	1999	Prospective case series	Denmark	12 to 24 months

Armenti, S.T., Miller, J.M.L., Gomez-Hassan, D., Gappy, C. & Cornblath, W.T.	2021	Retrospective case report	United States	1.5 years
	
Astle, W. & Miller, S.J.	1994		–	5 years
	
Biousse, V., Newman, N.J., Petermann, S.H. & Lambert, S.R.	2000		–	2 years
	
Defoort-Dhellemmes, S., Denion, E., Arndt, C.F., Bouvet-Drumare, I., Hache, J. & Dhellemmes, P.	2002		–	7 years
	
Fukuo, Y., Abe, T. & Hayasaka, S.	1998		–	1 year
	
Hussaindeen, J.R., Mani, R., Agarkar, S., Ramani, K.K. & Surendran, T.S.	2014		–	1 year
	
Kang, K.D., Kang, S.M. & Yim, H.B.	2006		–	1 year
	
Lee, J.M., Kim, S.H., Lee, J.I., Ryou, J.Y. & Kim, S.Y.	2009		Korea	More than 12 months


As summarised in Tables 5 and 6, participants were between three to 32 years of age. Each study reported on clinical features of every patient at initial presentation and follow-up post-treatment, including any refractive errors and prism cover test measurements of the deviation size at near and distance. Treatment outcomes and prognosis were determined in terms of cover test results in three patients, presence of binocular single vision (BSV) in 29 patients and a combination of both in seven patients.

**Table 5:** Summary of findings for benign risk factors.


BENIGN										

AUTHOR(S)	RISK FACTOR(S)	TYPE OF AACE	INCIDENCE	CLINICAL CHARACTERISTICS ON INITIAL PRESENTATION				TREATMENT	PROGNOSIS	
	
				AGE	GENDER	REFRACTION	DEVIATION SIZE OF ET (NEAR, DISTANCE)/Δ		DEVIATION SIZE ATFINAL PRESENTATION(NEAR, DISTANCE)/Δ	BINOCULAR FUNCTION

Hussaindeen, J.R., Mani, R., Agarkar, S., Ramani, K.K. & Surendran, T. S. (2014)	Accommodative spasm	Unclear	1	23	M	R. –1.00/+1.25 × 180 L. –1.75/+1.00 × 180	30, 30	Discontinuation of current overcorrected gls, New bifocals, Mydriatics (1% Atropine and 2% Homatropine), Accommodative facility exercises	Ortho, Ortho	–

Kang, K.D., Kang, S.M. & Yim, H.B. (2006)	Swelling or inflammation from orbital cellulitis	2	1	6	M	R. +1.00/+0.50 × 75 L. +0.75/+0.50 × 80	28, 22	BMRRec	Ortho, Ortho	Stereopsis 100”

Lyons, C.J., Tiffin, P.A.C. & Oystreck, D. (1999)	Uncorrected hypermetropia	Other – Refractive-accommodative	3	5	–	+3.75 OU	60, 35	Gls for full optical correction	–	Stereopsis 60”
	
				10	M	R. +2.25 L. +1.50	30, 30	Gls for full optical correction, BMRRec		Stereopsis 40”
	
				5	M	R. +4.00 L. +3.50	40, 35	Gls for full optical correction, BMRRec		Stereopsis 40”
	
	Monofixation syndrome	Other – Decompensating microtropia	4	3.5	–	+2.50 OU	20, 25	Gls for full optical correction		MF
	
				4	F	R. +2.75 L. +1.50	25, 25	Gls for full optical correction		MF
	
				24	F	R. +5.75 L. +4.75	30, 20	Gls for full optical correction, BMRRec		MF
	
				5	F	+2.25 OU	20, 20	Gls for full optical correction		MF
	
	Unclear	2	1	7.5	–	+1.50 OU	60, 50	Gls for full optical correction, BMRRec		Stereopsis 60”

Sefi-Yurdakul, N. (2022)	Excessive computer or smartphone use (≥4 hours a day)	3	6	7.5	F	R. +0.75 L. +1.00	50, 55	?Gls for full cycloplegic refractive error*, BMRRec	Ortho, Ortho	Stereopsis 100”

				5.5	M	R. +1.50 L. +2.00	30, 35	Gls for full cycloplegic refractive error, BMRRec	ET 2, ET 4	Stereopsis 100”

				4	M	R. +2.00 L. +2.00	40, 40	Gls for full cycloplegic refractive error, BMRRec	ET 10, ET 8	No BSV

				9	M	R. –2.00 L. –2.25	45, 40	Gls for full cycloplegic refractive error, LMRecss-LLResec	ET 4, ET 2	Stereopsis 100”

				8	M	R. –1.50 L. –1.50	40, 35	Gls for full cycloplegic refractive error, LMRecss-LLResec	ET 6, ET 2	Stereopsis 100”

				12	M	R. –2.25 L. –3.00	40, 35	Gls for full cycloplegic refractive error, LMRecss-LLResec	Ortho, Ortho	Stereopsis 100”

	Emotional stress	2	1	13	F	R. –2.75 L. –2.75	25, 30	Gls for full cycloplegic refractive error, BMRRec	ET 2, ET 2	Stereopsis 100”

	Monocular occlusion	1	1	5	M	R. +1.50 L. +1.75	35, 30	Gls for full cycloplegic refractive errorBMRRec	Ortho, Ortho	Stereopsis 100”

Simon, A.L. & Borchert, M. (1997)	Refractive	Other – Refractive-accommodative	1	7	M	R. +6.25 L. +4.00	40, 50	Gls for full cycloplegic refractive error	Orthophoria, Orthophoria	Stereopsis 40”
	
	Combined mechanism (Refractive + Other unknown cause)		3	5	M	R. +1.75/+0.75 × 90 L. +3.00/+0.50 × 90	55, 50	Gls for full cycloplegic refractive error, BMRRec	E(T) 12, E(T) 12	Stereopsis 50”

				5	F	R. +1.00 L. +1.00	70, 50	Gls for full cycloplegic refractive error, BMRRec	EP 5, EP 5	Stereopsis 140”

				8	M	R. +5.00/+0.25 × 40 L. +4.00/+0.25 × 180	55, 55	Gls for full cycloplegic refractive error, BMRRec	Orthophoria, Orthophoria	Stereopsis 40”

Turan, E.K. & Kansu, T. (2016)	Unclear	2	7	32	M	Between –1.50 to +0.50 OU	40, 40	RMRecss-RLResec	No manifest deviation	Stereopsis 100”
	
				29	M		16, 18	Prism	No manifest deviation (with prism)	Stereopsis 100”
	
				23	M		45, 45	Refused treatment	ET 45, ET 45	ISQ
	
				20	F		20, 20	Prism	No manifest deviation (with prism)	Stereopsis 100”
	
				22	F		25, 25	LMRecss-LLResec	No manifest deviation	Stereopsis 100”
	
				32	F		18, 16	Prism	No manifest deviation (with prism)	Stereopsis 100”
	
				27	F		35, 40	LMRecss-LLResec	No manifest deviation	Stereopsis 100”


Key: ET = Esotropia, E(T) = Intermittent esotropia, EP = Esophoria, M = Male, F = Female, R = Right, L = Left, OU = Both eyes, LE = Left eye, BMRRec = Bilateral medial rectus recession, LMRecess = Left medial rectus recession, LLResec = Left lateral rectus resection, Gls = Glasses, Ortho = Orthotropia, BSV = Binocular Single Vision, MF = Monocular fixation, W4LT = Worth 4 Light Test, ISQ = In status quo. *Unclear if glasses were prescribed for this patient as optical corrections were only given for refractive errors of one and more dioptres in the study.

**Table 6:** Summary of findings for non-benign risk factors.


NON-BENIGN											

AUTHOR(S)	RISK FACTOR(S)	TYPE OF AACE	INCIDENCE	CLINICAL CHARACTERISTICS ON INITIAL PRESENTATION					TREATMENT	PROGNOSIS	
	
				AGE	GENDER	REFRACTION	DEVIATION SIZE OF ET (NEAR, DISTANCE)/Δ	ADDITIONAL NEUROLOGICAL SIGNS		DEVIATION SIZE ATFINAL PRESENTATION (NEAR, DISTANCE)/Δ	BINOCULAR FUNCTION

Armenti, S.T., Miller, J.M.L., Gomez-Hassan, D., Gappy, C. & Cornblath, W.T. (2021)	Multiple sclerosis	Neurologic	1	16	M	–2.25 OU	20, 30	Headache, End-gaze jerk nystagmus	Neurological treatment, Prism for 1 year then weaned	EP 2, EP 2	–
	
Astle, W. & Miller, S.J. (1994)	Cerebellar tumour		1	15	M	Plano OU	45, 45	Worsening frontal and temporal headaches, Possible family history of brain tumour	Resection of tumour	EP 10–15, EP 10–15	Stereopsis 40”
	
Biousse, V., Newman, N.J., Petermann, S.H. & Lambert, S.R. (2000)	Arnold-Chiari Type 1 malformation		1	14	F	–	35, 35	–	Suboccipital decompression & laminectomy	Mainly orthophoric, EP 2 in extreme L gaze*	–
	
Defoort-Dhellemmes, S., Denion, E., Arndt, C.F., Bouvet-Drumare, I., Hache, J. & Dhellemmes, P. (2002)	Arnold-Chiari Type 1 malformation		1	9	M	+1.50 OU	30, 35	Frontal headache, Micro-nystagmus in all directions	Suboccipital decompression	EP 2, EP 4	Stereopsis 40”
	
Fukuo, Y., Abe, T. & Hayasaka, S. (1998)	Head trauma and high level of carbamazepine		1	11	M	R. +1.00 L. +1.25	40, 40	Convulsions	Reduce carbamazepine dosage to 400 mg/day	Ortho, Ortho	Stereopsis 120”
	
Lee, J.M., Kim, S.H., Lee, J.I., Ryou, J.Y. & Kim, S.Y. (2009)	Cerebellar Tumour		1	3	M	+0.75 OU	50, 50	–	Excision of tumour, Patching, BMRRec	ET 8, ET 8 – Was briefly ortho post-surgery for a month	No motor fusion
	
Lyons, C.J., Tiffin, P.A.C. & Oystreck, D. (1999)	Cerebellar Tumour		1	4.5	F	Plano OU	35, 35	–	Excision of tumour, BMRRec	–	Stereopsis 40”
	
Simon, A.L. & Borchert, M. (1997)	Arnold-Chiari Type 1 malformation		1	11	F	+0.50 OU	16, 20	Down-beating nystagmus, Difficulty walking, Poor balance	Neurological treatment, BMRRec	Orthophoria, XP 3	Diplopia on W4LT


Key: ET = Esotropia, EP = Esophoria, XP = Exophoria, M = Male, F = Female, R = Right, L = Left, OU = Both eyes, BMRRec = Bilateral medial rectus recession, Gls = Glasses, Ortho = Orthotropia, W4LT = Worth 4 Light Test. *Given the comitancy of esotropia and presence of full ocular movements at initial presentation, the authors support the diagnosis of AACE though a mild left sixth nerve palsy could have also been a possible differential diagnosis.

### Study outcome

Findings from each included study were categorised and presented as benign (Table 5) and non-benign risk factors (Table 6) for AACE. An overview of the incidence, AACE subtype, clinical characteristics, treatment and prognosis of each reported patient were also summarised in the respective table.

## Discussion

### Findings

#### Benign risk factors

As outlined in Table 5, a wide range of benign risk factors for AACE was presented across the studies with the exception of Turan and Kansu (2016). Within each case series, the most common risk factor found varied from decompensating microtropia (Lyons, Tiffin and Oystreck, 1999) to excessive near work (Sefi-Yurdakul, 2022) to a combination of hypermetropia and other undetermined causes (Simon and Borchert, 1997). Other less reported risk factors included accommodative spasm (Hussaindeen et al., 2014), orbital cellulitis (Kang, Kang and Yim, 2006), emotional stress and binocular disruption (Sefi-Yurdakul, 2022).

When considering the three AACE subtypes proposed by Burian and Miller (1958), benign risk factors were most commonly associated with Type 2 AACE followed by Types 3 and 1. However, besides inflammation and stress as identified in Kang, Kang and Yim (2006) and Sefi-Yurdakul (2022) respectively, most risk factors for Type 2 AACE were unknown as reported in Lyons, Tiffin and Oystreck (1999) and Turan and Kansu (2016). In addition to the original three subclassifications, AACE of decompensating microtropia and refractive-accommodative subtypes have also been suggested in Lyons, Tiffin and Oystreck (1999) and Simon and Borchert (1997) with the identified risk factors being microtropia and uncorrected hypermetropia respectively.

Comparing the clinical characteristics of AACE reported in each paper, hypermetropia was the most common refractive error noted in patients with all being below +3.00 diopters besides in cases of refractive-accommodative AACE (Lyons, Tiffin & Oystreck 1999; Simon & Borchert 1997). Myopia was found to be as equally common in patients with Type 3 AACE (Sefi-Yurdakul, 2022). In general, both near and distance deviation sizes averaged around 35Δ for all AACE subtypes except for decompensating microtropia which had a smaller mean size of 23Δ. Treatment varied according to the AACE subtype. For patients with either Type 1 or 3 AACE, glasses for refractive errors of one and more dioptres were prescribed as first-line treatment before strabismus surgery was performed to correct any remaining deviation (Sefi-Yurkadul, 2022). For patients with Type 2 AACE, full optical corrections were given at first visit in Lyons, Tiffin and Oystreck (1999) and Sefi-Yurkadul (2022). However, due to the non-accommodative characteristic of this subtype, all patients still required either prisms or surgery to regain binocularity (Kang, Kang and Yim, 2006; Lyons, Tiffin and Oystreck, 1999; Sefi-Yurkadul, 2022; Turan and Kansu, 2016). For all patients with refractive-accommodative and decompensating microtropia AACE, refractive errors were first fully corrected with glasses before surgery for any remaining esotropia was conducted (Lyons, Tiffin and Oystreck, 1999; Simon and Borchert, 1997). For the patient with accommodative spasm-induced AACE, new bifocals based on the full cycloplegic refraction was prescribed in conjunction with mydriatics and orthoptics exercises to relax accommodation (Hussaindeen et al., 2014). Good prognosis for each AACE subtype was reported across all studies with patients demonstrating and maintaining a minimum of gross stereopsis throughout the duration of follow-up besides Sefi-Yurdakul (2022), though the reason for this outcome was not discussed. With the exception of Lyons, Tiffin and Oystreck (1999), all studies also documented stable final deviation sizes of less than or equal to 12Δ of esotropia if not orthotropic. There was no change in signs and symptoms in the patient who refused treatment in Turan and Kansu (2016).

#### Non-benign risk factors

As outlined in Table 6, Arnold-Chiari Type 1 malformation (Biousse et al., 2000; Defoort-Dhellemmes et al., 2002; Simon and Borchert, 1997) and cerebellar tumour (Astle and Miller, 1994; Lee et al., 2009; Lyons, Tiffin and Oystreck, 1999) were most commonly found to be associated with neurologic AACE followed by single case reports of multiple sclerosis (Armenti et al., 2021) and head trauma and carbamazepine (Fukuo, Abe and Hayasaka, 1998).

As a whole, in contrast to the reported clinical features of benign AACE, no particular refractive status was more frequently identified than the other in neurologic AACE. While deviation sizes averaged similarly around 35Δ, additional neurological signs were noted with nystagmus (Armenti et al., 2021; Defoort-Dhellemmes et al., 2002; Simon and Borchert,1997) and headaches (Armenti et al., 2021; Astle and Miller, 1994; Defoort-Dhellemmes et al., 2002) being the most common. All studies presented a management plan involving initial neurological treatment, in which Astle and Miller (1994), Biousse et al. (2000), Defoort-Dhellemmes et al. (2002) and Fukuo, Abe and Hayasaka (1998) found was sufficient to re-establish orthotropia and BSV in patients. Additional prisms were given in Armenti et al. (2021) to alleviate symptomatic diplopia and were successfully weaned off as the patient regained orthotropia after commencing long-term antibody therapy. In cases whereby strabismus surgery was performed, stereopsis was regained post-operation in Lyons, Tiffin and Oystreck (1999). Fusion remained absent in Simon and Borchert (1997) despite an orthotropic post-operative ocular alignment and in Lee et al. (2009) where esotropia recurred a month post-surgery.

### Strengths and limitations

This review was limited by the inclusion of only case series and reports, both of which are low-level research evidence, with small sample sizes. The generalisability of findings were further restricted as most articles failed to mention a study location. Additionally, given the retrospective design of most studies and absence of controls in all studies, the quality of clinical data presented was reduced, preventing any establishment of a reliable cause-effect relationship between the identified risk factor(s) and AACE.

This review was also limited by its narrow inclusion criteria, resulting in only a few articles being included despite a robust search strategy. Restricting full-text articles to English also prevented the analysis of all pertinent data on the risk factors for AACE. The need for a minimum one-year follow-up duration excluded a few case-control studies specifically investigating the risk factors for Type 3 AACE, which findings could have provided greater insight into the relationship between near work, myopia and AACE development with greater reliability as compared to the retrospective case series by Sefi-Yurdakul (2022) where recall bias and subjectivity of ‘excessive close work’ were present.

While this review was conducted in a well-structured manner with all included papers being comprehensive and of high quality, caution should still be taken when applying the findings from this study due to the reduced significance and generalisability of results.

### Contextualisation of findings

Current available knowledge on the pathogenesis and types of AACE remains limited with most information being derived from small-scale case series and reports. While individual papers such as Lyons, Tiffin and Oystreck (1999) and Simon and Borchert (1997) have suggested new AACE subclassifications in addition to the three subtypes defined by Burian and Miller (1958), these were isolated proposals that were unsubstantiated by other papers. With Lyons, Tiffin and Oystreck (1999) proposing a decompensating microtropia subtype based on the final features of binocular function in patients’ post-treatment, more prospective research following patients with known microtropia and tracking of any subsequent AACE development could be conducted first to better understand the incidence and mechanism of decompensating microtropia before considering it as a subclassification. The need to include refractive-accommodative subtypes under AACE is also arguable as accommodative esotropia is presently a well-established, independent esotropia subtype in current literature.

That said, findings from this review support the notion of neurologic AACE as a rare and distinct subtype though its definitive ophthalmic characteristics were unclear. Despite near-distant disparities of over 40% being noted as a sign of neurologic AACE in Buch and Vinding (2015), no significant size differences were recorded in the included studies besides Armenti et al. (2021). With the lack of other obvious neuro-ophthalmic and/or neurological signs in Biousse et al. (2000), Lee et al. (2009) and Lyons, Tiffin and Oystreck (1999), the need for neuroimaging in all patients with AACE could be justified. Nonetheless, given the low incidence rates of neurologic AACE, the feasibility and cost-effectiveness of performing these scans remain debatable.

The heterogeneity of our study findings supports the elusiveness of AACE. However, due to low population and geographical generalisability, no definite conclusion on the significance of each identified risk factor can be drawn and future research with larger-scale prospective case-control or cohort studies is required. In particular, given the rise of AACE cases in recent years (Wu et al., 2020), independent risk factors for Type 3 AACE such as excessive close work as identified in Sefi-Yurdakul (2022) require further validation via more rigorous study designs with objective measurements and longer follow-up periods to allow clinicians to better advise and manage patients with Type 3 AACE.

Despite uncertainties in the classification of and risk factors for AACE, similarities in the identified clinical features, treatment and prognosis for each respective AACE subtype were found in this review. Additional studies could be conducted to ascertain the value of these clinical signs as diagnostic and/or prognostic factors and evaluate the effectiveness of these treatment options, facilitating clearer and more standardised reporting of AACE cases. With an increased insight, current guidelines could then be continually enhanced to improve future practise in the management of patients with AACE.

## Conclusion

This review has provided a brief overview on the possible risk factors for and clinical features of AACE in children and young adults and supports the proposal of neurologic AACE as a new, though rare, subclassification. However, higher-level research evidence is required to better substantiate any cause-effect relationship, determine the significance of devising additional benign AACE subtypes and establish the value of neuroimaging in AACE cases before solid conclusions and advice can be provided.
